# An innovative light chamber for measuring photosynthesis by three-dimensional plant organs

**DOI:** 10.1186/s13007-018-0288-5

**Published:** 2018-03-14

**Authors:** A. Fortineau, P. Bancal

**Affiliations:** UMR ECOSYS, INRA, AgroParisTech, Université Paris-Saclay, 78850 Thiverval-Grignon, France

**Keywords:** Quantum yield, Photosynthesis, Gas exchange, Three-dimensional organs, Light distribution, Wheat, Grapevine, Fruit, Ecology

## Abstract

**Background:**

In plants, three-dimensional (3-D) organs such as inflorescences or fruits carry out photosynthesis and thus play a significant role in carbon assimilation and yield. However, this contribution has been poorly characterized because there is no reliable method for measuring photosynthesis by 3-D organs. One of the major challenges is ensuring the uniform irradiation of samples that are placed within a sealed chamber.

**Results:**

In this study, we developed an innovative chamber with homogeneous lighting that can be used to measure photosynthesis by large 3-D organs. It consisted of a 15-cm-long sealed transparent cylinder that was surrounded by a decagonal prismatic light source, made up of a mixture of red and blue LEDs. We characterized irradiance homogeneity within the chamber at a resolution level of 1 cm and 10°. Photosynthetic photon flux density (PPFD) along the central axis of the chamber could be set to any value between 100 and 1100 µmol m^−2^ s^−1^. The coefficient of variation for the irradiation values found throughout the chamber was 10% and that for the ratio of red-to-blue spectra was less than 1.5%. The temperature of the sample was regulated to stay within 1 °C of the target temperature, regardless of PPFD. We compared the performance of our device with that of a commercially available device employing unidirectional lighting. Specifically, we examined net photosynthesis in two sample types—wheat ears and grape clusters—at varying PPFD levels.

**Conclusions:**

The devices gave similar estimates of dark respiration, regardless of sample type or age. Conversely, net photosynthesis started to become asymptotic at lower irradiance levels in our device than in the conventional device because apparent quantum yield was three times higher. When examining the effects of irradiance heterogeneity, it was clear that biased estimates could result from systems employing unidirectional light sources. Our results also confirmed that our chamber could be a useful tool for obtaining more accurate estimates of photosynthesis by 3-D organs.

**Electronic supplementary material:**

The online version of this article (10.1186/s13007-018-0288-5) contains supplementary material, which is available to authorized users.

## Background

Photosynthesis is the main bioenergetic process used by plants. It is carried out in chlorophyll-containing cells: carbon dioxide (CO_2_) taken in from the air through the stomata is converted into carbohydrates using light energy, while water is transpired through the same stomata. It is necessary to fully understand photosynthesis if efforts to predict and improve the quantity and quality of crop yields have to succeed. Higher-quality data on photosynthesis would be of great interest to those in the wine and agricultural industries. They would also be helpful in the field of ecotoxicology, namely in the development of pollution or stress biomarkers, and could help inform fundamental and applied research. At present, portable photosynthesis analyzers are often employed to assess net photosynthesis, intercellular CO_2_ concentrations, stomatal conductance, and transpiration. The results can be used to characterize leaf lamina functionality in relation to genotype and/or environmental conditions, revealing plant phenotypes.

Leaf laminas are the plant organs largely responsible for photosynthesis, and it is frequently possible to quantify dynamics at the crop level from data obtained at the lamina level. For example, Cabrera-Bosquet et al. [1] demonstrated that, in wheat, photosynthesis levels in flag-leaf laminas could be used to infer photosynthesis levels in the entire crop. Consequently, most whole-plant and crop models are based on photosynthesis by laminas, as that of Farquhar et al. [2], even though the contributions of other photosynthetic organs, as the stem [3, 4] and fruits [5], have largely been recognized. If canopy models that neglect non leaf organs work, it is mostly because of the allometric factors existing between leaves and canopy, but often to the detriment of a setting that does not comply with reality. For example, we measured the absorption coefficient of the wheat leaves (0.35) which is only half that of the canopy (0.67). At a time when this parameter becomes a phenotyping criterion, such an approximation can mask varietal behaviors of interest and make selection difficult.

However, fully characterizing the contribution of different plant organs to overall photosynthesis is crucial, especially because relative contributions may shift. For example, lamina area declines under conditions of drought, grazing, or foliar disease, which means that photosynthesis by other organs takes on greater importance. Furthermore, fruit and seed numbers are key components of crop yield. In fruit trees, these numbers are dependent on circumstances during early development—nutrition has a well-established role [6]. Indeed, the autotrophic level of photosynthesis by green fruits is a variable of interest [5].

As most photosynthesis analyzers have been designed for use with planar organs, such as laminas, the devices employ unidirectional lighting, which is not appropriate when dealing with three-dimensional (3-D) organs such as fruits. Since the rate of photosynthesis is not directly proportional to irradiance levels, thick organs must be entirely and homogeneously illuminated to avoid having to average full-light and shade responses. Moreover, many 3-D organs are also large, which means they have higher rates of respiration; this fact can have a significant impact on net photosynthesis. Consequently, respiration in large 3-D organs cannot be neglected, as it often is for laminas. Last, but not least, the relative levels of gross photosynthesis and respiration likely fluctuate during fruit development and may depend on environmental conditions (e.g., temperature, source/sink balance). Studies taking into account 3-D organs often arrive at contradictory results. Kreidemann [7] used different combinations of shading techniques and gas-exchange measurements to assess the photosynthetic contribution of wheat ears during the grain-filling period. Since that study, others have attempted to do the same, but the resulting ear contributions have varied from 10 to 40% or more, depending on the nature of the study and the methods used [8, 9]. It would therefore appear that photosynthesis cannot be fully characterized at the crop level without a better understanding of the contribution made by large 3-D organs.

Several studies have attempted to address the problem of light homogeneity when measuring photosynthesis by such organs. For example, Idle and Proctor [10] built a 150-mm-diameter integrating sphere to ensure that plant organs experienced homogeneous irradiance. However, the system’s physics resulted in a trade-off between sphere volume and plant size. In smaller spheres, the distribution of light was not uniform, leading to measurement error, but in larger spheres, the light available to the plant was reduced. Their device attained a photosynthetic photon flux density (PPFD) of 300 µmol m^−2^ s^−1^, which is not high enough to maximize the rate of photosynthesis at saturating irradiance (A_max_) in most terrestrial plants. Ireland et al. [11] modified the lighting system so that it reached 800 µmol m^−2^ s^−1^. However, this PPFD could only be achieved using a smaller sphere (100 mm in diameter), which meant that it could not be used with large samples. When light-emitting diodes (LEDs) were developed in the early 1990s, a revolution took place in photosynthesis-related lighting. Tennessen et al. [12] extolled the reliability and portability of LED-based systems, as well as the repeatability of the data they produced. They demonstrated that red 665-nm LEDs resulted in photosynthesis-irradiance curves that were similar to those obtained using white xenon lamps with PPFDs of 1000 µmol m^−2^ s^−1^. Nowadays, this form of lighting is widely used in photosynthesis measurement devices. For example, Sanchez-Bragado et al. [13] built a customized chamber for wheat ears that was fully enclosed and that had an external LED-based light source. This device could attain a PPFD of 1200 µmol m^−2^ s^−1^, which allowed A_max_ to be achieved. However, the light source also heated samples to 30 °C, a warm temperature that crops rarely experience. Moreover, neither the system’s spectral quality nor its irradiance homogeneity was characterized. Hogewoning et al. [14] clearly demonstrated that irradiance homogeneity is critical in ensuring accurate measurements of photosynthesis.

In this study, we developed a device for estimating photosynthesis by large 3-D organs; the goal was to achieve homogeneous irradiance and limited organ heating. Using a mixture of red and blue LEDs, the device reached a PPFD of 1100 µmol m^−2^ s^−1^. We designed the apparatus for indoor and outdoor use and made it compatible with a commercially available photosynthesis analyzer (the LI-6400XT; LI-COR, Lincoln, Nebraska, USA). We evaluated the homogeneity of the lighting conditions and the degree of sample warming. We also used the device to measure net photosynthesis in two sample types—wheat ears and grape clusters. We compared the results with those obtained using a conventional device with a unidirectional light source.

## Material

### Device description

**Fig. 1:**
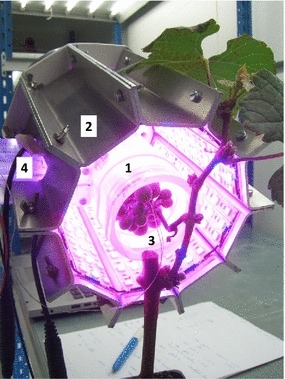
Sample chamber (1) surrounded by the prismatic light source (2). A thermocouple (3) measures the temperature of the sample, which is a grape cluster. There is a hole through which tubing is passed, connecting the sample chamber and the infrared gas analyzer (IRGA) (4). For illustrative purposes, the back panel of the system is removed

The apparatus was composed of two parts: (i) a transparent cylindrical sample chamber surrounded by (ii) a prismatic light source (Fig. 1). The device was designed to be portable and battery powered; it was intended for use in both the laboratory and the field.

The cylindrical sample chamber was made of a transparent poly(methyl methacrylate), or PMMA, tube. Since PMMA adsorbs water [15], the inner face of the chamber was coated with polytetrafluoroethylene (PTFE) tape to minimize water sorption/desorption. The upper end of the tube was sealed with a PMMA plug. A mixing fan (50 L min^−1^) was attached to the plug such that it faced downward into the chamber. The bottom end of the tube was where the sample could be introduced. The chamber was connected to the sensor head of a LI-6400XT system (LI-COR, Lincoln, Nebraska, USA); its position was offset to allow for the prismatic light source. The tubing that connected the chamber to the infrared gas analyzer (IRGA) was as short and direct as possible to take advantage of the sensor head’s Peltier cooler and mixing fan. This design allowed us to balance device shape, volume, and air flow in such a way as to allow adequate air mixing and cooling. The air in the chamber (volume of 300 cm^3^: 160 mm high and 50 mm in diameter) could be renewed every 20 s when the IRGA’s maximum flow rate (700 µmol s^−1^) was used. Such a flow rate was needed to limit both water condensation and the warming of the air and the sample. Taking into account the IRGA’s sensitivity to CO_2_ differentials (0.15 vpm), a minimum net carbon exchange rate of 0.1 nmol CO_2_ s^−1^ per organ was needed.

Because leaks can have a major impact on gas-exchange measurements, photosynthetic chambers must be completely sealed [16]. We created a seal by placing a cylindrical latex membrane around the bottom of tube; the membrane was clamped to the sample’s stem using a clothespin. Leak rates in the chamber were then quantified as described by Flexas et al. [17]: a heat-killed wheat ear, i.e., without any gas exchange that would influence the results, was inserted in the chamber. Then a combination of flow rates and CO_2_ concentrations in IRGA reference (Cr) was applied. The match valve was systematically used for each combination. The CO_2_ concentration in the lab (Ce) was continuously measured using Li-840A (Li-cor, Lincoln, Nebraska, USA). The diffusions of CO_2_ between the lab and the chamber modified the concentration of CO_2_in the chamber (Cs), leading to a difference (Cs–Cr) which resulted in an apparent net photosynthesis (aPn) different from zero. According to the Li-6400 manual [18], the true net photosynthesis (Pn) is deduced from aPn using Eq. (1):

1 $${\text{Pn}} = {\text{aPn}} + {\text{k}} \cdot \left( {{\text{Ce}} - {\text{Cs}}} \right) / {\text{S}}$$

providing both aPn and Pn are indicated in µmol CO_2_ s^−1^ m^−2^, both Ce and Cs in µmol mol^−1^, with S being the sample area (in mm^2^) and k a normalized diffusion coefficient.

The prismatic light source was made of aluminum sheets and surrounded the sealed chamber. The prism was decagonal; each face was a rectangle of 30 mm by 150 mm. The dimensions were dictated by the size of the individual LED strips (Colasse SA, Seraing, Belgium). Each rectangle was covered with three strips: two red (660 nm) strips were placed on either side of a blue (450 nm) strip.

The LEDs mounted on linear strips (SMD: Surface mounted diode) had a 120° beam angle. Their emission patterns were Lambertian, which means that the irradiance profile was proportional to the cosine of the emission angle. This fact promoted light homogeneity in the prism’s center. The PPFD level inside the chamber was monitored using a mini quantum sensor (LSC, Walz, Effeltrich, Germany), which was located near the tubing that connected the chamber and the IRGA. This quantum sensor was plug to the IRGA’s BNC connector and thus yielded quantitative measurements of PPFD during the gas-exchange analyses.

PPFD levels significantly decreased when the temperature of the LEDs exceeded 40 °C. The outer faces of the light source were equipped with aluminum fins that served as heat sinks. In addition to this passive cooling system, an exhaust fan (80 mm in diameter; NMB technologies, Chatsworth, California, USA) was attached to the interior of the prism’s top panel. It generated a vertical flow of air upwards (rate of 1.0 m^3^ min^−1^) through the empty space between the sealed sample chamber and the light source. At ambient temperature (around 20 °C), the temperature of the light source remained below 40 °C even when the LEDs were run at maximum levels for extended periods of time.

Irradiance levels could be adjusted using one of the IRGA’s digital-to-analog ports. An analog converter (Serem, Saint-Remy-de-Maurienne, France) was used to transform the 0–5 VDC output to 0–10 VDC signal input in order to regulate the LED controller (LINEARDrive 720, eldoLED, Eindhoven, Netherlands), which powered the light source’s 264 LEDs. It should be noted that six LEDs were removed to create the passage for the tubing that linked the sample chamber and the IRGA, which resulted in an issue with irradiance (see Results).

### Light homogeneity within the sample chamber

To characterize the spectral distribution inside the sample chamber, a miniaturized cosine corrector sensor (1800-11 remote cosine receptor; LI-COR, Lincoln, Nebraska, USA) was attached to an optic fiber, which was itself linked to a spectroradiometer (UniSpec; PP-Systems, Amesbury, Massachusetts, USA) with a resolution of 3.3 nm that measured spectra in the 300–1100 nm range. The sensor’s sensitive side was oriented towards the inner face of the light source. The sensor was attached to a rotary axis with a graduated handwheel, which allowed the sensor to rotate 360° in the chamber’s center. The rotary axis was fastened to a linear translation stage. Using both the rotary axis and the translation stage, we could obtain accurate 3-D spectral images with resolutions of 10° and 1 cm, respectively. The light gradient in the radial direction was also quantified, displacing the sensor by 5 mm increments along the diameter from one inner face of the chamber to the other. All the data were analyzed using Qua^2^Ray software [19]. The PPFD measurements were used to calibrate the mini quantum sensor inside the chamber.

### Photosynthesis measurements

The output from our prototype device, hereafter referred to as the P-chamber, was compared with that from a commercially available device equipped with a unidirectional light source. This latter device, hereafter referred to as the C-chamber, was a LI-COR 6400-22L Opaque Conifer Chamber equipped with a 6400-18A RGB Light Source. It was designed to measure net photosynthesis by 3-D organs within a 330-cm^3^ sample chamber. For both devices, the concentration of incoming CO_2_ was maintained at 400 μmol mol^−1^ using an LI-6400-01 CO_2_ injector employing a high-pressure liquid CO_2_ cartridge. Air temperature and relative humidity were kept at 25 °C and 60%, respectively. Gas exchange was measured at the following PPFD levels: 1000, 500, 200, 100 and 0 μmol m^−2^ s^−1^ for the P-chamber and 2000, 1000, 500, 100 and 0 μmol m^−2^ s^−1^ for the C-chamber. When the plant samples were used (see below), measurements were taken at each PPFD level after approximately 10 min had passed—once CO_2_ and stomatal conductance had stabilized. Organ temperature was measured using a type E (nickel-chromium/constantan) thermocouple that was clamped either between two spikelets located in the middle of the wheat ear or at the back of a grape berry. Both chambers also measured air temperature and atmospheric pressure.

### Plant samples

Net photosynthesis was measured for two types of plant samples: (i) ears of wheat (*Triticum aestivum* L., cv. Soissons) and (ii) clusters of grapes (*Vitis vinifera* L., cv. Pinot Noir).

The larger dimensions of the P-chamber meant that it could handle samples up to 15 cm long, and thus most wheat ears. However, the C-chamber could only handle samples that were 8 cm or less in length, which meant that it could not fit regular-sized ears (the Soissons variety has awns). We therefore used small ears that were produced by extending the photoperiod during plant development (i.e., from sowing to maturity): the longer days hastened plant development but resulted in smaller ears. First, wheat seeds were vernalized by exposing them to a temperature of 5 °C and a photoperiod of 16 h for 6 weeks. They were then transplanted into pots containing loam and left in a greenhouse where PPFD was 275 μmol m^−2^ s^−1^ and the photoperiod lasted 16 h. Wheat anthesis began on October 23, 2015. Net photosynthesis by the wheat ears was measured twice: on November 5 or 6 and on November 23 or 24.

Fruiting cuttings were taken from Pinot Noir canes in accordance with the procedure described in Lebon et al. [20]. The cuttings were then planted in pots containing loam and placed in a growth chamber kept at 25 °C (both day and night) and 60% relative humidity; there was a 16-hour photoperiod, and PPFD was 300 μmol m^−2^ s^−1^. Grapevine flowering began on September 10, 2015. Net photosynthesis by the grape clusters was measured twice: on October 5 or 6 and on October 19 or 20.

### Surface area of the photosynthetic organs

We carefully measured the projected and developed areas of the photosynthetic organs because these areas are important when calculating photosynthetic parameters. 3-D scanning is a promising approach, but it was not available with the required accuracy in our lab at the time the experiment was done. Instead, the length and two widths of wheat ears were measured (± 0.1 mm), yielding estimates of both projected area and developed area; the ears were treated as parallelepipeds, and the awns were not taken into account. The grape clusters were irregularly shaped, and the berries displayed varying degrees of overlap. Consequently, the numbers of berries were counted. Then, the total projected area of each cluster of berries was determined via image analysis. By dividing this projected area by berry number, we obtained a projected diameter that was smaller than the true diameter because of berry overlap. This projected diameter was then used to calculate the developed areas of berries, which were treated as spheres. The same approach was then used for each cluster’s stalk, which was treated as a cylinder. The developed area of a cluster was the sum of the developed areas of its berries and of its stalk.

## Results

### Photon flux densities and spectral distributions

To control the irradiance level, the relationship between PPFD (μmol m^−2^ s^−1^) and the input voltage (U) was determined. The quantum sensor placed in the center of the chamber indicated that PPFD was linearly correlated with input voltage. However, there was a small offset since the LEDs turned on at values above 1.10 VDC and turned off at values below 0.95 VDC. However, this offset did not result in hysteresis. For higher voltages (up to 10 VDC), the relationship between PPFD and input voltage could be described by the following equation:

2 $${\text{PPFD}} = 103.18*{\text{input}}\;{\text{voltage}} - 64$$

The coefficient of determination for this relationship was very high (r^2^ > 0.999). With our light source, it was possible to set the PPFD level inside the chamber to any value between 100 and 1100 µmol m^−2^ s^−1^.

The red and blue LEDs had slightly different responses to input voltage. Consequently, the relative contribution of the red LEDs (600–700 nm) to PPFD (range of 400–700 nm) shifted as the input voltage changed: it was 64.2% at 10 VDC but 67.2% at ~ 1 VDC.

**Fig. 2:**
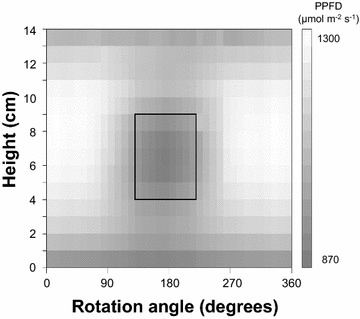
360° representation of irradiance in the chamber as measured from its center. Each pixel represents a different angle (x axis; 10° resolution) and height (y axis; 1 cm resolution). The scale on the right indicates the PPFD level (μmol m^−2^ s^−1^) in gray (voltage input: 10 VDC)

There was heterogeneity in PPFD within the chamber (Fig. 2); the coefficient of variation (CV) reached 10%. Irradiance was reduced at the top and the bottom of the chamber. Furthermore, in Fig. 2, there is a 5 cm by 90° rectangle representing a central shaded area in which PPFD was 18% lower than elsewhere. This shaded area accounted for about 11% of the total area (Table 1) and was due to the six LEDs that were removed to allow for the tubing that connected the sample chamber and the IRGA.

**Table 1:** Irradiance heterogeneity within the chamber

	Total area (100%)	Fully lit area (89%)	Shaded area (11%)
Mean PPFD at 10 VDC (μmol m^−2^ s^−1^)	1133 ± 114	1160 ± 95	949 ± 47
Ratio of mean PPFD at 10 VDC to mean PPFD at 2 VDC	6.807 ± 0.142	6.808 ± 0.146	6.794 ± 0.109
Proportion of red light at 10 VDC	0.634 ± 0.009	0.633 ± 0.008	0.647 ± 0.005

The means (± standard deviation) for the total area are compared with those of the fully lit (89% of total) and shaded (11% of total) areas

Measurements were similar for the three voltage levels tested (2, 5, and 10 VDC). Also, the proportion of red light (relative to all PPFDs) was quite stable throughout the chamber across voltage levels (CV < 1.5%). Furthermore, the shaded area always appeared in the same position and was always of the same magnitude at the three voltage levels.

We also confirmed that there was no significant spectral heterogeneity. Across the entire chamber, the coefficient of variation for the proportion of red light at 10 VDC was always lower than 2%. While the shaded area experienced significantly redder light (*P *< 10^−4^; Table 1), the magnitude of the difference was likely too small to have a significant biological effect. The results were similar for different voltages: the coefficient of variation for the proportion of red light across voltage levels was less than 1%. These findings thus suggested that both the quantity and quality of light within the chamber were highly homogeneous regardless of voltage level.

The objects in the chamber are extending beyond the central axis, and moreover they could be imperfectly aligned on this axis. The light gradient in radial direction was thus quantified: In most cases, the PPFD increases by 5.5% from the central axis when getting 1 cm closer to the inner illuminating surface of the device, and it decreases at the same rate when getting away from it. By exception, when the sensor faced the central shaded area, getting 1 cm closer to the inner, shaded, surface of the chamber decreases PPFD by 8.7%. Thus the irradiance heterogeneity will increase for bigger samples in the chamber.

### Response time and leak rate

**Fig. 3:**
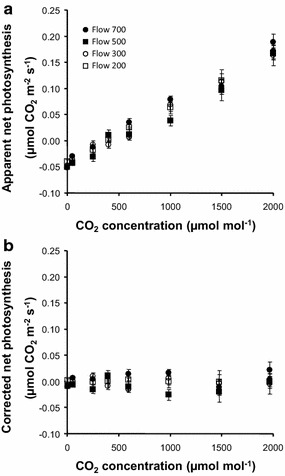
Leakage quantification. An oven dried ear with a developed area of 43.2 cm^2^ was inserted in the chamber which was then flushed with various CO_2_ concentrations and flow rates. Leakage modified CO_2_ concentration in the chamber, and the difference with the set CO_2_ concentrations resulted in an apparent net photosynthesis (**a**). The later was corrected according to Eq. (1) with a diffusion coefficient k = 0.45: the corrected net photosynthesis (**b**) was nil at any CO_2_ concentration and flow rate. Values are the average ± standard deviation of six measurements

To assess response time, the flow rate in the chamber was 700 µmol s^−1^, the maximum allowed by the analyzer. To determine the re-equilibration delay when settings were changed, the CO_2_ concentration was increased from 100 to 400 μmol mol^−1^, and CO_2_ levels were monitored in the IRGA’s reference and sample chambers. There was a 38-s delay between the two IRGA chambers to reach 385 μmol mol^−1^ CO_2_ concentration (or 95% of the step-in setting level). This delay could reflect the physical time needed to reach a steady state. Plant samples need far more time to adjust following such a switch; during our measurements, samples required a 10-min stabilization period. Thus, the flow rate in the chamber was high enough to prevent delayed re-equilibration when settings were changed. Leak rate was then estimated. Setting the chamber with CO_2_ concentrations lower than outside the chamber led to a positive difference between the reference and the chamber. Conversely the difference was negative when the chamber was set with higher CO_2_ concentrations. It resulted in an apparent net photosynthesis, according to Eq. (1). Since the CO_2_ concentration in the surrounding lab (Ce) is quite constant, the apparent net photosynthesis should linearly correlate with CO_2_ concentration in the chamber (Cs). Such pattern was indeed observed using flow rates higher than 200 µmol s^−1^ (Fig. 3a), but not for lower flow rates, underlining the fact that the chamber is too big to be properly flushed by small flow rates. Given that the true net photosynthesis (Pn) by killed ear should not correlate with Cs, the diffusion coefficient k of the chamber was fitted in order for the slope of Pn to (Ce–Cs) to be nil (Fig. 3b). The obtained value (k = 0.448 ± 0.009) shows the leakage in our chamber is not higher than in the commercially available leaf chambers. Under normal conditions of use (i.e., Cs is close to Ce), the leakage error would be less than 0.05 µmol CO_2_ m^−2^ s^−1^, this value being less than 1% of Pn for a living ear such as that in Fig. 3b. So, similarly to what is suggested for commercially available chambers, we can conclude that the leakage of our chamber can be neglected under normal conditions of use.

### Temperature regulation

**Fig. 4:**
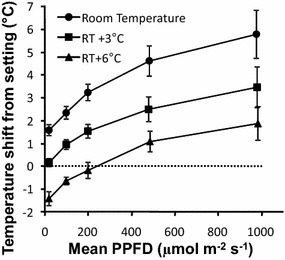
Temperatures experienced by an ear of wheat in the sample chamber. The air conditioning temperature was set either at room temperature, or 3 °C higher, or 6 °C higher. The difference between mean sample temperature and the temperature of the incoming air are plotted depending on PPFD level. The error bars represent the standard error associated with thermocouple measurements made at various positions on an ear triplicate

The analyzer was equipped with an air conditioning system that was not designed for use with high-powered light sources. When the empty chamber was experiencing maximum irradiance, the air temperature was 1.5 °C higher than the temperature setting, even when the maximum flow rate was used. Moreover, this result was obtained by setting the temperature to room temperature. The chamber warmed less when a warmer setting was employed. Taken together, these results suggest that the cooling capacity of the air conditioning system was overwhelmed under high-irradiance conditions. No spatial heterogeneity in air temperature was detected inside the empty chamber, but the results were different when a plant sample was present in the chamber (Fig. 4). When a room-temperature setting was used, wheat ear temperature was 2–6 °C higher, depending on irradiance levels. When the air conditioning temperature was set to 6 °C above room temperature, wheat ear temperature ranged within ± 2 °C of that temperature set, depending on irradiance levels. The effects of irradiance on wheat ear temperature were counteracted by placing the device in a growth chamber that was 5 °C cooler than the target temperature and by varying the analyzer’s temperature setting based on PPFD. This approach allowed us to achieve thermal stability (± 1 °C) at any irradiance level. Nevertheless, the temperature varied along the wheat ear’s length: its bottom end was 3 °C warmer than its top end, which accounts for the increasing error bars in Fig. 4.

### Impact of heterogeneous irradiance

**Fig. 5:**
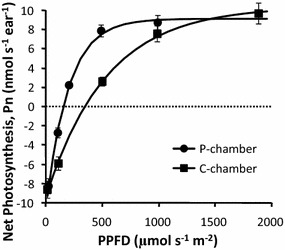
Photosynthetic responses by wheat ears at different irradiance levels in the P-chamber and the C-chamber. Net photosynthesis (P_n_) in wheat ears that were 2 weeks post anthesis was measured at different PPFD levels in the P-chamber and the C-chamber. Ear temperature was always 25 °C. The means and standard deviations were calculated from four ears with a mean developed area of 22.3 cm^2^. The lines are described by the Eq. 3

When using conventional devices with unidirectional lighting, such as the C-chamber described above, responses for the fully lit and shaded parts of the sample are averaged. The P-chamber was built to obtain more holistic estimates of photosynthesis. When we compared the results for the C-chamber and the P-chamber, we found some major differences. For example, when net photosynthesis (P_n_) in wheat ears was tested at a constant 25 °C ear temperature 2 weeks after anthesis, the results were very similar in the dark and at maximum irradiance but differed strongly at intermediate light levels (Fig. 5). Indeed, it could be argued that the very meaning of irradiance was not comparable between the devices. Therefore contrary to what is frequently done, we will not express measurements per square meter of organ area. We could have expressed measurement per gram of fresh weight, but when fast growing organs like fruits are concerned, this unit is somewhat misleading. Finally, since our objective is to compare two devices, we measured ears of the same size and we expressed net photosynthesis in nmol s^−1^ per ear rather than in µmol s^−1^ per unit area (for the reader convenience, Additional file 1: Figure S1 plots P_n_ in µmol s^−1^ m^−2^ and in nmol s^−1^ gFW^−1^).

A total of 24 wheat ears and 24 grape clusters were measured. Data obtained from each plant sample were first fitted via least-squares minimization to the following equation:

3 $${\text{P}}_{\text{n}} \left( {\text{I}} \right) = {\text{A}}_{\text{max} } \cdot \left[ {1{-}\exp \left( { -\upalpha \cdot {\text{I/A}}_{\text{max} } } \right)} \right]{-}{\text{R}}_{\text{d}}$$

where P_n_(I) is net photosynthesis at irradiance level I and A_max_, α, and R_d_ are maximal assimilation at saturating irradiance (when CO_2_ = 400 µmol mol^−1^), apparent quantum yield, and dark respiration, respectively. R^2^ was always greater than 0.95. The parameter values fitted using P-chamber data are referred to as A_p_, α_p_, and R_p_, and those fitted using C-chamber data are A_c_, α_c_, and R_c_. Because we used two sample types and because samples varied in age, the parameters had a wide range of values. However, the values from P-chamber were similar to those from C-chamber under conditions of darkness and saturating irradiance. For instance, when we fitted the wheat-ear data (Fig. 5), we found that A_p_ = 18.7 ± 1.3 nmol s^−1^ ear^−1^ while A_c_ = 19.6 ± 1.9 nmol s^−1^ ear^−1^ and that R_p_ = 9.5 ± 0.9 nmol s^−1^ ear^−1^ while R_c_ = 9.1 ± 0.9 nmol s^−1^ ear^−1^. In contrast, photosynthesis became light saturated at a lower irradiance level in the P-chamber than in the C-chamber. Ninety-nine percent of A_max_ was reached at a PPFD of 1000 μmol m^−2^ s^−1^ in the P-Chamber, while 2000 μmol m^−2^ s^−1^ was needed to reach 97% of A_max_ in the C-chamber (Fig. 5). At intermediate levels of irradiance, net photosynthesis was much greater in the P-chamber than in the C-chamber, which resulted in very different values for α (α_p_ = 0.042 ± 0.001 mol CO_2_ mol photon^−1^ vs. α_c_ = 0.015 ± 0.001 mol CO_2_ mol photon^−1^ for the data depicted in Fig. 5). To further compare the two chambers, we calculated the relative root mean square errors (RRMSEs) for all the parameter estimates obtained from the P-chamber and the C-chamber (Table 2, row 1).

**Table 2:** Relative root mean square errors (RRMSEs; in %) for estimates of the parameters A_max_, α, and R_d_ obtained from the P-chamber and C-chamber

	Maximal assimilation at saturating irradiance A_max_	Apparent quantum yield α	Dark respiration R_d_
Simple equations	6%, 23%	167%, 200%	9%, 19%
Equations accounting for irradiance heterogeneity	15%, 21%	13%, 28%	6%, 18%

In the first row are the RRMSEs for the estimates obtained by directly fitting the photosynthesis raw data. In the second row are the RRMSEs for the estimates obtained after rewriting the equations to account for irradiance heterogeneity. In each case, the first percentage is associated with the wheat ears and the second with the grape clusters

A_max_ ranged from 9 to 20 nmol s^−1^ organ^−1^ for the wheat ears and from 0.5 to 3 nmol s^−1^ organ^−1^ for the grape clusters. R_d_ ranged from 4 to 10 nmol s^−1^ organ^−1^ for the wheat ears and from 1 to 3 nmol s^−1^ organ^−1^ for the grape clusters. The RRMSEs were acceptable for A_max_ and R_d_; they were lower for the wheat ears than for the grape clusters (Table 2) because of the increased sensitivity of the high measurement values. Conversely, the estimates of α clearly differed based on whether the P-chamber or the C-chamber was used. Indeed, there may have been a device-related bias because, regardless of sample characteristics (i.e., sample type or age), α_c_ was about one-third of α_p_ (Fig. 5).

 To check if the discrepancy was due to irradiance heterogeneity, the data were fitted a second time, taking into account that, in the C-chamber, only part of the sample was fully lit. According to the information provided by the manufacturer, the rest of the sample was exposed to a PPFD level equivalent to 0.25 I. The C-chamber data were thus fitted using the following equation:

4 $${\text{P}}_{\text{n}} \left( {\text{I}} \right) = {\text{S}}_{\text{p}} / {\text{S}}_{\text{d}} \cdot {\text{A}}_{\text{max} } \cdot \left[ {1{-}\exp \left( { -\upalpha\cdot{\text{I/A}}_{\text{max} } } \right)} \right] + \left( {1{-}{\text{S}}_{\text{p}} / {\text{S}}_{\text{d}} } \right) \cdot {\text{A}}_{\text{max} } \cdot \left[ {1{-}\exp \left( { -\upalpha \cdot 0.25 \cdot {\text{I/A}}_{\text{max} } } \right)} \right]{-}{\text{R}}_{\text{d}}$$

where S_p_ and S_d_ are the projected and developed areas of the sample, respectively. The same approach was used with the P-chamber data because, as discussed above, about 11% of the sample was in the shaded area (Fig. 2) and was exposed to 0.86 I; the fully lit part of the sample received 1.05 I. The P-chamber data were thus fitted using the following equation:

5 $${\text{P}}_{\text{n}} \left( {\text{I}} \right) = 0.89 \cdot {\text{A}}_{\text{max} } \cdot \left[ {1{-}\exp \left( { -\upalpha \cdot 1.05 \cdot {\text{I/A}}_{\text{max} } } \right)} \right] + 0.11 \cdot {\text{A}}_{\text{max} } \cdot \left[ {1{-}\exp \left( { -\upalpha \cdot 0.86 \cdot {\text{I/A}}_{\text{max} } } \right)} \right]{-}{\text{R}}_{\text{d}}$$

**Fig. 6:**
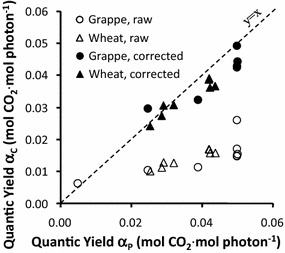
Apparent quantum yields estimated by fitting data from the P-chamber (α_p_) and the C-chamber (α_c_). Each point corresponds to a plant sample, either a wheat ear or a grape cluster. The estimates of apparent quantum yields (mol CO_2_ mol photon^−1^) were either calculated directly from the raw photosynthesis data or corrected using equations that take into account irradiance heterogeneity. The line depicts a one-to-one relationship between α_p_ and α_c_

The final results suggested that irradiance heterogeneity could be ignored when the P-chamber was used because α_p_ decreased only 2.4% for both sample types while A_p_ and R_p_ demonstrated a change of less than 0.1%. Conversely, α_c_ tripled when irradiance heterogeneity was accounted for (Eq. 4 vs. Equation 3). Moreover, the corrected estimate of α_c_ was similar to α_p_ (Table 2 and Fig. 6). It can be concluded that the raw data from the C-chamber greatly underestimated α. A more accurate estimate of α could only be obtained by correcting for irradiance heterogeneity or by using the P-chamber.

## Discussion

Commercially available field photosynthesis analyzers are made to be used with leaf laminas. Because they employ unidirectional light sources, they are not adapted to 3-D samples, such as fruits. Yet, there is an increasing need to study photosynthesis by such organs to improve our understanding of plant physiology, to promote disease resistance and tolerance in plants, and to help plant species confront global warming.

In this study, we built an innovative photosynthesis measurement device that was designed to be used with 3-D organs. It was composed of a prismatic light source coupled with a commercially available field analyzer. It obtained accurate measurements of net photosynthesis by large 3-D organs at different levels of irradiance thanks to its design; the system uniformly lit the organs while simultaneously preventing them from overheating.

Irradiance levels in the sample chamber could be controlled and spanned a range from 100 to 1100 µmol m^−2^ s^−1^. When irradiance levels throughout the chamber were assessed, it was found that the coefficient of variation was less than 10%, which means that the lighting was fairly homogeneous [14].

Because of the light source’s configuration (i.e., it enclosed the sample chamber), the introduction of any sample was certain to have an impact on irradiance. Once illuminated all around by the lighting device, it is to be noted that, when the sample does not stand on the chamber central axis, it receives a little less light from one side and more light from the opposite side: imperfect centering has little consequence. On the other hand, the bigger the samples are, the closer they get to the LEDs and the more light they receive. However, it is not recommended to introduce samples larger than 2.5 cm in diameter in a chamber of 5 cm diameter since it could disturb the air flow. According to measurements of radial light gradient, such a sample would receive a slight 7% over-irradiance. Wheat ears fit the chamber well because they are essentially simple cylinders with small radii. Consequently the irradiance variability between the ears measured (where diameter variability is less than 1 cm) will only be of a few percent. In contrast, grape clusters display more complex geometry, and it was unlikely that each berry was exposed to the same level of irradiance. This limitation resulted from the size ratio between the sample and the chamber. For larger samples, a larger chamber should be used, so as to decrease the variability in distance between the LEDs and the different parts of the sample. However, such a change can only be achieved at the expense of irradiation level. This trade-off was observed in previous experiments carried out with spherical light sources [10]. While this limitation must be acknowledged, we nonetheless propose that our device represents an improvement over conventional devices because we confirmed that irradiance levels were more homogenous in our sample chamber than in a conventional chamber with unidirectional lighting.

We also compared the performance of our device—the P-chamber—with that of a conventional chamber—the C-chamber—using two types of plant samples: wheat ears and grape clusters. For both chambers, maximal assimilation at saturating irradiance (A_max_) and dark respiration (R_d_) were similar. Since the lighting was more uniform in the P-chamber, net photosynthesis started becoming asymptotic at 1100 µmol m^−2^ s^−1^. In the C-chamber, with its unidirectional lighting, this value was 2000 µmol m^−2^ s^−1^. Conversely, net photosynthesis was significantly higher in the P-chamber than in the C-chamber at intermediate irradiance levels. When we corrected for the heterogeneous light conditions in the C-chamber (Eq. 3), it was clear that this heterogeneity ultimately led to inappropriate averaging of the photosynthetic response between the fully lit and shaded areas. The correction resulted in reasonable estimates of net photosynthesis when the latter was modeled using three parameters (i.e., A_max_, α, R_d_). However, more complex models, such as the one proposed by Farquhar et al. [2], would not be able to use data obtained from a C-chamber. They could, though, take advantage of the more holistic data obtained from the P-chamber. The P-chamber gets closer to measuring photosynthetic capacity as tissues are illuminated from all angles rather than just from one direction. It gives a better understanding of what the tissue is capable of achieving (which may be useful for breeders) but does not measure what the organ achieves in the field, where sunlight is partly directional (although including at least 20% of diffuse radiation) and organs routinely self-shaded. In a one-sided lighting device, the fully directional irradiance is not natural but any sample will be self-shaded in a similar way and therefore results will be repeatable although not controlled. The homogeneous irradiance in P-chamber is clearly not natural, but it is controlled. Not only is it possible to achieve physiological unbiased measurements, but it may also be possible to calculate the photosynthesis of the organs under natural conditions thanks to the use of 3-D simulations to estimate the irradiance per unit area.

The quality of light inside the chamber was also of key interest. To minimize power consumption and radiation heating, we built our P-chamber using red and blue LEDs in a 2:1 ratio, which constrained light input to the spectrum needed to optimize photosynthesis. Red and blue wavelengths were chosen because (i) McCree [21] characterized maximum photosynthetic yield within red and blue wavelengths; (ii) Zieger and Hepler [22] showed that blue light plays an important role in stomatal aperture control; and (iii) Shimazaki et al. [23] reviewed how light regulates stomatal opening and suggested that blue light and red light act in synergy. Given this information, it is possible that spectral heterogeneity could result in a single organ displaying mixed responses (e.g., only some of its stomata close). For this reason, we examined the distribution of light spectra inside the chamber. The coefficient of variation was less than 2%, which means that any significant physiological effects were unlikely. Spectral homogeneity could have easily been obtained using white LEDS. However, it was important to confirm that it could be created using different colored LEDs because it would be interesting to equip the chamber with RGB LEDs to study the effect of light spectra on photosynthesis by 3-D organs.

Lighting also has a significant impact on temperature. If temperature is not carefully regulated, the light source can warm the plant sample via radiative heat transfer. It is crucial to control sample temperature since it has disparate effects on the activity of respiration and photosynthetic enzymes [24]. Indeed, respiration hardly reaches 10% of gross photosynthesis in leaf laminas; therefore the bias resulting from uncertainties concerning the measurement of the respiration only amounts to a few percent of net photosynthesis. In large 3-D organs where the respiration rate could be twice that of the rate of the gross photosynthesis, both respiration and net photosynthesis should be accurately taken into account. Furthermore, photosynthesis and respiration are unlikely to exhibit a constantly proportional relationship in response to growth stage, stress, and genotype. For this reason, the temperature of large 3-D organs must be closely monitored and controlled.

When we designed our device, we made every attempt to limit conductive heat transfer from the light source to the sample chamber by ventilating the intervening space. The dimensions of the tubing that connected the sample chamber to the IRGA were chosen to take advantage of the analyzer’s air conditioning system. In the LI-6400XT, a type E thermocouple is used to measure organ temperature and adjust the cooling system’s efforts accordingly (i.e., the temperature setting refers to desired organ temperature). This basic system could not be used with the P-chamber because the analyzer’s air conditioning system was overwhelmed. Consequently, we had to work in a growth chamber where the ambient temperature was 5 °C below the desired organ temperature. For different ambient temperatures and irradiance levels, we determined the relationship between the temperature setting for the analyzer’s air conditioner and the temperature of the plant sample. This method allowed us to modify the temperature set according to irradiance in order to tightly control organ temperature (± 1 °C). However, this solution severely hampers system portability. Ultimately, it would be better to regulate temperature using an additional thermoelectric cooling system positioned upstream from the sample chamber; we are currently working on a design.

Finally, temperature homogeneity across the sample is as important as irradiance homogeneity within the chamber. This issue was not comprehensively addressed because doing so would require placing many thermocouples on the sample, which would impair air circulation and irradiance homogeneity. While temperature regulation is clearly the device’s main weakness, our results have shown that using a flawed device with uniform lighting is still better than using a device with unidirectional lighting. Moreover, temperature homogeneity could also be increased via improvements in the air conditioning system.

To the best of our knowledge, our device is the first of its kind: a chamber dedicated to measuring photosynthesis by 3-D organs that displays irradiance homogeneity and limited sample heating. It can also measure stem photosynthesis because the sample is placed in a cylindrical chamber. The light source and sample chamber can be associated with commercially available photosynthesis analyzers, making both laboratory and field measurements theoretically possible.

In closing, Flexas et al. [17] warned against the effects of leakages on photosynthesis parameterization. They quantified the diffusion coefficient of an empty leaf chamber at 0.46, which is close to 0.44 predicted by the manufacturer [18]. However they observed the leak rate was modified when inserting dead sample between the gaskets of the chamber. Moreover, this rate was varying between each sample, suggesting the leakage occurred between the gaskets where the sample is clamped. Since the sample is fully inserted into our chamber, the diffusion coefficient will vary only slightly with its stem perimeter, whatever the sample measured and regardless of the plant species. The value of 0.45 that we observed is therefore fair enough to make our chamber a suitable tool for studying the physiology of the photosynthesis by 3D plant organs. On the other hand, some more conceptual modelling work is needed to assess internal CO_2_ concentration for large organs. Indeed, the commonly employed Farquhar equations [2] rely on internal CO_2_ concentration that cannot be directly measured. But nevertheless, the internal CO_2_ concentration in leaves can be quantified by using models that estimate CO_2_ diffusion from the air to chloroplasts. In large organs, however, a significant amount of the internal CO_2_ is released by the respiration, making photosynthesis rate somewhat depending on the diffusion from respiration sites. Modifying the Farquhar model so that it could be applied to large organs is however far beyond the scope of this paper.

## Conclusions

Our results show that our device provides reliable estimates of net photosynthesis for two types of 3-D plant organs: wheat ears and grape clusters. We discovered that the estimates obtained with our device, which used homogeneous lighting, were less biased than those obtained with a conventional device, which used unidirectional lighting. Furthermore, our device can be used to assess rate and efficiency of both photosynthesis and respiration. The information it can provide on CO_2_ and water vapor exchange parameters could be used to analyze stomatal conductance and the other factors limiting photosynthetic performance. The results presented above clearly illustrate the device’s potential as a powerful tool that could be used to improve our understanding of the relationships between plant structure and physiology. Any topics that need information about gas exchanges by non-leaf organs could benefit from such device.

## Additional file

**Additional file 1:** Figure S1. Photosynthetic responses by wheat ears at different irradiance levels in the P-chamber and the C-chamber. Net photosynthesis is reported either to full organ, thus in nmol CO_2_ s^−1^ per ear, as in text (A), or to photosynthetic area, thus in µmol CO_2_ s^−1^ m^−2^ (B), or to biomass, thus in nmol CO_2_ s^−1^ gFW^−1^ (C).

## Abbreviations

3-D: Tri-dimensional
PPFD: Photosynthetic photon flux density
IRGA: Infra-red gas analyzer
LED: Light emitting diode
PMMA: Poly(methyl methacrylate)
PTFE: Polytetrafluoroethylene
P_n_: Net photosynthesis
A_max_: Maximal assimilation at saturating irradiance
α: Apparent quantum yield (mol CO_2_ mol photons^−1^)
R_d_: Dark respiration
RRMSE: Relative root mean square errors
