# Supplementary material for: An innovative light chamber for measuring photosynthesis by three-dimensional plant organs
Source: Plant Methods. 2018 Mar 14;14:21. doi: 10.1186/s13007-018-0288-5 (PMC5853119; doi:10.1186/s13007-018-0288-5)
Supplement: Supplementary file 1 — Additional file 1: Figure S1. Photosynthetic responses by wheat ears at different irradiance levels in the P-chamber and the C-chamber. Net photosynthesis is reported either to full organ, thus in nmol CO2 s−1 per ear, as in text (A), or to photosynthetic area, thus in µmol CO2 s−1 m−2 (B), or to biomass, thus in nmol CO2 s−1 gFW−1 (C). [file 13007_2018_288_MOESM1_ESM.docx]

SUPPLEMENTARY DATA

***Figure S1: Photosynthetic responses by wheat ears at different irradiance levels in the P-chamber and the C-chamber.***

*Net photosynthesis (P_n_) in wheat ears that were two weeks post anthesis was measured at different PPFD levels in the P-chamber (circles) and the C-chamber (squares). Ear temperature was always 25°C. The means and standard deviations were calculated from four ears with a developed area of 22.3 ±0.9 cm² and a fresh weight of 1.64 ±0.15 g. The lines are described by the equation* P_n_(I) = A_max_·[1 – exp(-α ·I/A_max_)] – R_d_*. , where P_n_(I) is net photosynthesis at irradiance level I and A_max_, α, and R_d_ are maximal assimilation at saturating irradiance (when CO_2_ = 400 µmol mol^-1^), apparent quantum yield, and dark respiration, respectively.*

*Net photosynthesis is reported either to full organ, thus in nmol CO_2_ ∙s^-1^ per ear, as in text (A), or to photosynthetic area, thus in µmol CO_2_ ∙s^-1^ ∙m^-2^ (B), or to biomass, thus in nmol CO_2_ ∙s^-1^ ∙gFW^-1^ (C).*

| *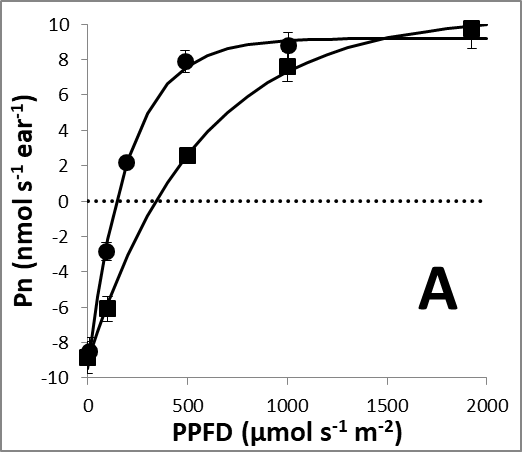* | *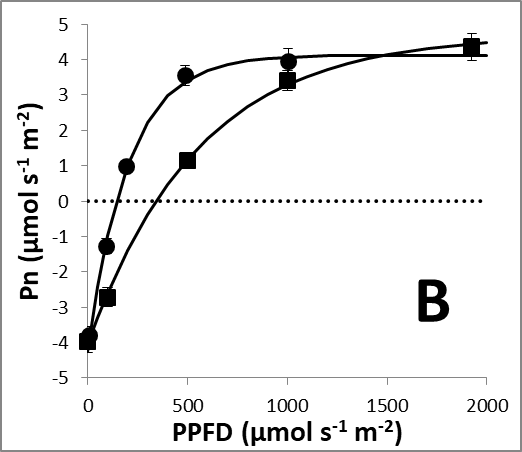* | *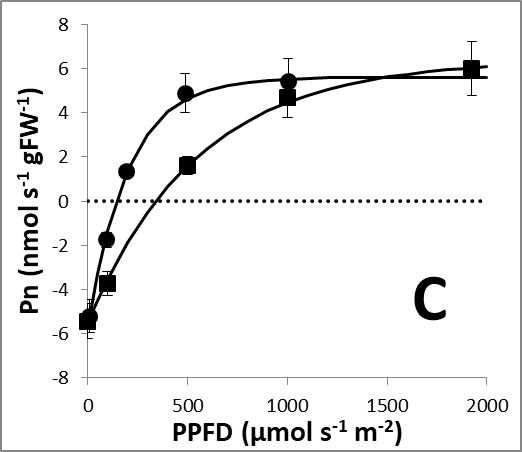* |
| --- | --- | --- |
